# Terrestrial Establishment of Strangler Figs in Burned Tropical Peat Swamp Forests, Sumatra, Indonesia

**DOI:** 10.1002/ece3.73194

**Published:** 2026-02-27

**Authors:** Sidiq Purwanto, Muhammad Iqbal, Douglas Sheil

**Affiliations:** ^1^ Forest Ecology and Forest Management Chair Group Wageningen University and Research Wageningen the Netherlands; ^2^ Restorasi Ekosistem Riau, Jalan Lintas Timur, Pangkalan Kerinci Kabupaten Pelalawan Riau Indonesia; ^3^ Ecology and Forest Management Group, Department of Environmental Sciences Wageningen University Wageningen the Netherlands; ^4^ Center for International Forestry Research Kota Bogor Indonesia

**Keywords:** *Ficus*, peat‐swamp‐forest, restoration, wetlands

## Abstract

Strangler figs (*Ficus* spp.) typically establish as epiphytes on host trees before developing ground roots; terrestrial establishment also occurs but is less widely recognised. Here we document abundant terrestrial establishment of strangler figs in burned tropical peat swamp forests of Sumatra, Indonesia. Across 3.4 ha of transects in two post‐fire sites, 97% of 138 strangler fig individuals (representing 10 species) germinated directly on the ground rather than epiphytically. *Ficus sundaica* dominated, comprising 22% of all trees and 67% of all figs. We find that strangler figs exhibit striking terrestrial establishment ability in degraded peatlands where active planting efforts have largely failed. Given their abundance, flexible establishment mode, and role as keystone species supporting frugivore communities, naturally regenerating strangler figs may facilitate broader ecosystem recovery in fire‐damaged peat swamp forests.

## Graphical abstract

Strangler figs (*Ficus* spp.) typically establish as epiphytes on host trees. Here we document abundant terrestrial establishment of strangler figs in burned tropical peat‐swamp forests of Sumatra, Indonesia. Given their abundance, flexible establishment mode, and role as keystone species supporting frugivore communities, naturally regenerating strangler figs may facilitate recovery in these ecosystems.

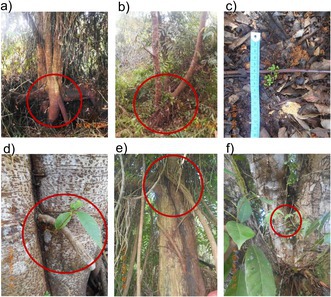

## 1 Introduction

Strangler figs (*Ficus* spp., Moraceae) are a distinctive and ecologically important category of trees. Their life cycle typically begins with epiphytic germination and establishment on a host tree, followed by root extension to the ground and eventual development into a free‐standing tree that may envelop and kill the host (Putz and Holbrook 1989). Due to their year‐round fruiting and role in supporting frugivores and related wildlife, and other ecological interactions, strangler figs are often considered keystone species (Shanahan et al. 2001; Weiblen 2002; Corlett 2006; Harrison 2005).

While typically described in terms of their epiphytic establishment, strangler figs occasionally colonise on other substrates and structures such as rock outcrops (Gates 1982) and walls and buildings (Jim 2014). Many strangler figs are also capable of terrestrial establishment (Zotz et al. 2021; Mo et al. 2022)—though ecologically this appears to be a neglected phenomenon. Abundant terrestrial establishment by strangler figs has not been reported as an important process in recovery of degraded forests. Here we report such a process in Indonesian peat swamp forests.

Tropical peat swamp forests have global significance, storing considerable carbon and supporting unique biodiversity (Posa et al. 2011). In Southeast Asia, many peat swamp forests have been degraded by drainage, clearing, and fires (Page et al. 2009), prompting considerable attention to their restoration (Tata and Pradjadinata 2016). Much restoration effort has focused on tree planting, as natural regeneration is often limited, though the survival of planted seedlings is also often poor (Puspitaloka et al. 2021).

Here we document the abundant terrestrial establishment of strangler figs in degraded (drained and burned) peatlands on the Kampar Peninsula, Sumatra, Indonesia. This represents, to our knowledge, the first systematic evidence of this phenomenon in peat swamp forests on this scale. One of us (MI) had previously noted that certain figs were strikingly common among the limited woody regrowth in some previously degraded areas where most ongoing restoration efforts were unsuccessful. As such, regeneration is desirable for restoration and seemed to occur directly on the ground; we were curious to verify, document and assess this phenomenon. Our findings highlight the ecological flexibility of strangler figs and show that they can indeed contribute significantly to natural regeneration in these degraded peatlands through terrestrial establishment.

## 2 Methods

Research was conducted in the Restorasi Ekosistem Riau (RER) concession on the Kampar Peninsula, Riau Province, Sumatra. This region holds some of Indonesia's deepest peat deposits (> 10 m) and extensive forests (Sari et al. 2021). In 2000, the peninsula lacked extensive human impacts but by 2014, 53.4% of the peatlands, mostly around the edge, had been drained and converted to plantations (pulpwood and some oil palm) while much of the forest had been selectively logged and impacted by canals and ditches. The drainage canals lowered the water table and left the remaining forest vulnerable to subsidence, drought and fire (Hooijer et al. 2015; Bell 2016). Since 2016, there have been restoration efforts actively closing canals, restoring the water table, slowing drainage, and promoting forest regrowth and recovery.

We selected two sites for study: Makmur (38 ha) and Sangar (30 ha), located 3.8 km apart.

Before the fires, Makmur likely served as a logging camp, with remnants like hoses and excavator parts found during firefighting in 2013, while Sangar had active illegal logging until 2012 and possibly had also served as a log yard. Fires in 2013–2014 left large open areas dominated by ferns and grasses interspersed with remnant forest. Makmur burned twice (in 2013 and 2014), while Sangar burned once (in 2013).

Restoration efforts began with canal blocking in August 2017, followed by enrichment planting with native seedlings collected from surrounding forest in August 2017, February 2018, April 2018, and September 2018. Establishment success was poor, and large open areas remained at the time of this study (June 2025) and served as the focus of our observations.

### 2.1 Site Characteristics

During the wet season both areas flood, while in the dry season low water tables leave the peat soils dry and flammable. The Sangar site, positioned close to the Sangar River (5–8 m wide), experiences prolonged annual flooding lasting 3–4 months with depths of 1–1.5 m extending up to 500 m into the forest. The Makmur site, surrounded by canals (2–4 m wide), is believed to experience briefer and shallower annual flooding.

Our observations, conducted shortly after flooding receded (following approximately three months of inundation), revealed that litter and soil remained moist in areas near canals at Makmur. Open areas near the river at Sangar were dominated by ferns with few woody plants.

We found that at Makmur, soil pH ranged from 4.5 to 5.5 where strangler figs were recorded, with litter thickness ranging from 0 to 12 cm. At Sangar, soil pH ranged from 3.5 to 5.0 and litter thickness from 0 to 8 cm.

### 2.2 Data Collection

We established 34 transects (100 × 10 m) across both sites: 19 in Makmur and 15 in Sangar. Transects were positioned using a stratified random approach and oriented west–east or east–west to capture environmental gradients from waterways and open burned areas into remnant forest. These transects avoided the occasional trees that had resulted from the past (largely failed) plantings as these occurred in lines that were still apparent.

Within each transect, we recorded all tree individuals ≥ 50 cm tall. Species were identified by collecting herbarium vouchers and consultation with experts. Soil pH and litter thickness were measured at three locations for each transect and averaged (at 0, 50, and 100 m).

For each strangler fig individual, we recorded: species identity; substrate and germination position (ground, tree fork, crevice, trunk); height and diameter; and rooting form (soil‐rooted vs. aerial host‐dependent). To distinguish naturally regenerated figs from any potentially planted individuals, we consulted RER planting records and staff familiar with past planting activities. Planted trees had been propagated from cuttings, positioned in regular 5 m × 5 m spacing patterns, and marked with coloured ribbons. Naturally regenerated figs germinated from seed (evidenced by taproot systems rather than adventitious rooting), did not coincide with known planting lines, and spanned a wide range of sizes including recent seedlings clearly established after planting activities ceased.

### 2.3 Classification of Establishment Mode

Each strangler fig individual was classified based on field observations of root networks and germination positions. Host‐dependent (epiphytic) establishment was defined as germination on a host plant with roots descending along the host before reaching soil. Host‐independent (terrestrial) establishment was defined as cases where the fig's root network reached the soil directly without wrapping around a host plant, with germination occurring on or near the ground. We recorded germination location using four categories: (1) ground, (2) forks in trees, (3) crevices on host tree, and (4) on branches or trunks. Implied germination height was measured using a Vertex hypsometer. While we had anticipated potentially difficult cases (such as figs growing over dead trees that may have fallen after establishment), all classification decisions proved simple and unambiguous in the field.

## 3 Results

### 3.1 Species Composition and Abundance

Among our 34,000 m^2^ (3.4 ha) of transects, we recorded 427 trees representing 35 species. Strangler figs accounted for 10 species and 138 individuals (32.3%). In open areas, strangler figs were abundant. The most common species overall was *Ficus sundaica* Blume (93 individuals, 21.8% of all trees), especially dominant in Makmur (81 individuals) (Table 1). Other common species included *Austrobuxus nitidus* Miq. (44 individuals, 10.3%), *Campnosperma coriaceum* (Jack) Hallier f. (41, 9.6%), *Diospyros siamang* Bakh. (32, 7.5%), *Syzygium zeylanicum* (L.) DC. (29, 6.8%), *Macaranga pruinosa* (Miq.) Müll.Arg. (21, 4.9%), *Ficus sumatrana* Blume (15, 3.5%), *Lithocarpus ewyckii* (Korth.) Rehder (15, 3.5%), *Shorea teysmanniana* Dyer ex Brandis (15, 3.5%), and *Ficus tristaniifolia* Corner (Berg & Corner) (13, 3.0%). By site, Makmur was dominated by *F. sundaica* (81), *D. siamang* (19), *S. teysmanniana* (15), *C. coriaceum* (14), *L. ewyckii* (14), *A. nitidus* (12), *Melicope lunu‐ankenda* (Gaertn.) T.G.Hartley (10), and *Sterculia gilva* Miq. (10). Sangar was dominated by *A. nitidus* (32), *C. coriaceum* (27), *M. pruinosa* (21), *S. zeylanicum* (21), *D. siamang* (13), and *F. sundaica* (12). Tree sizes ranged from seedlings to individuals with diameters at breast height up to ~45 cm and heights varying considerably.

**TABLE 1:** Counts of naturally regenerating strangler figs recorded in two burned peatland sites, Makmur and Sangar, Kampar Peninsula, Sumatra.

Species	Site			Establishment	
	Makmur	Sangar	Total	Epiphytic	Ground
*Ficus sundaica* Blume	81	12	93	✓(3)	✓
*Ficus sumatrana* Miq.	10	5	15		✓
*Ficus tristaniifolia* Corner	7	6	13		✓
*Ficus spathulifolia* Corner	0	6	6		✓
*Ficus retusa* L.	5	0	5		✓
*Ficus curtipes* Corner	0	2	2		✓
*Ficus caulocarpa* Miq	1	0	1		✓
*Ficus consociata* Blume	1	0	1		✓
*Ficus glaberrima* Blume	0	1	1	✓(1)	
*Ficus xylophylla* Wall. ex Miq.	0	1	1		✓
Total	105	33	138		

### 3.2 Establishment Mode

**FIGURE 1:**
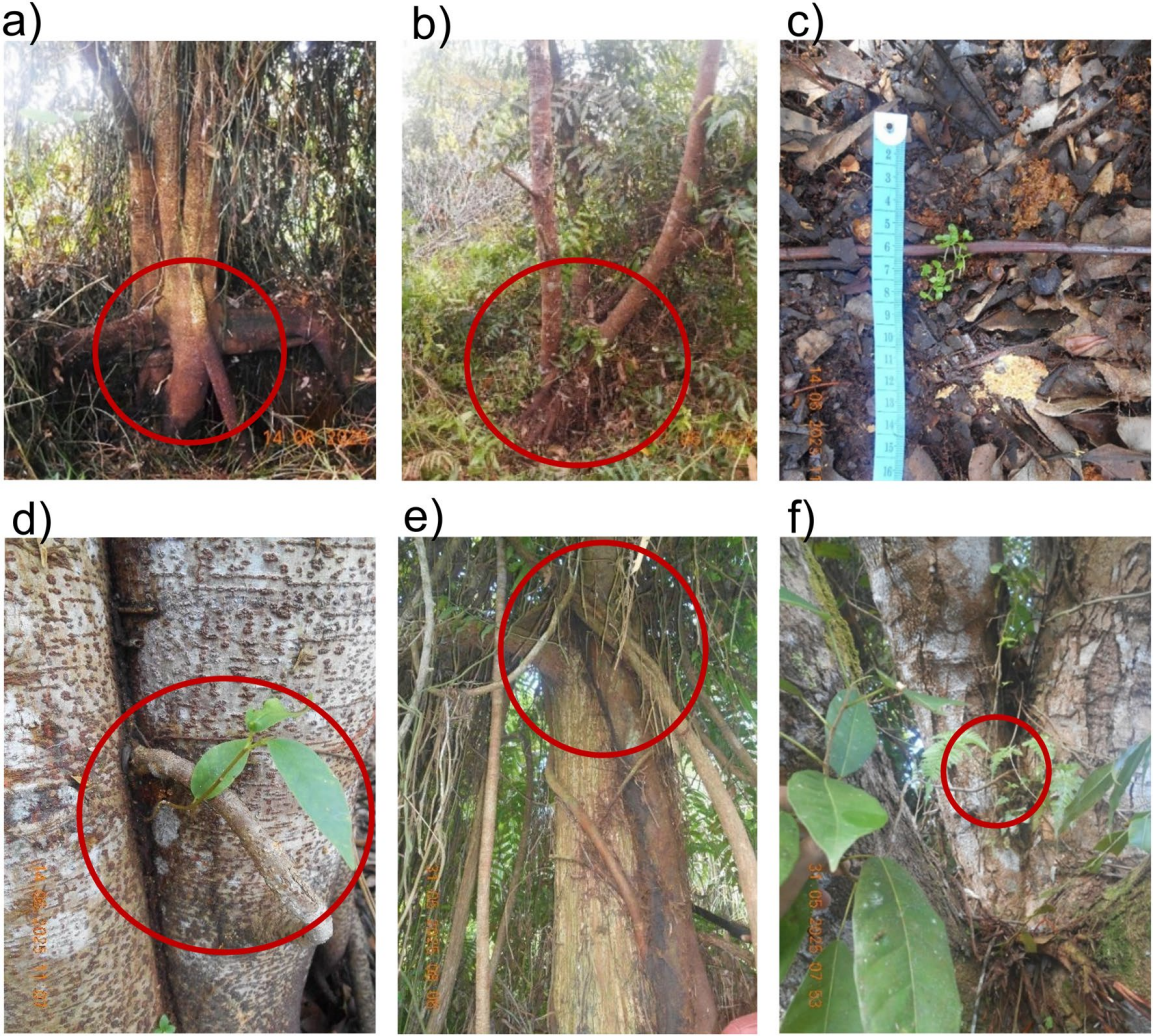
Strangler figs showing terrestrial and epiphytic establishment modes in burned peat swamp forest, Kampar Peninsula, Sumatra. Red circles indicate germination points. (a) Terrestrial *Ficus tristaniifolia* showing soil root system without aerial roots; (b) terrestrial *F. sundaica*; (c) recently germinated terrestrial *F. sundaica* seedlings; (d) epiphytic *F. sundaica* with roots descending along host trunk; (e) established epiphytic *F. sundaica*; (f) epiphytic *F. glaberrima* germinating in tree fork. Photographs: Sidiq Purwanto.

Of the 138 fig individuals, 134 (97.1%) established directly on or near the ground, rooted in soil and litter substrates (Figure 1). Only four individuals (2.9%)—three *Ficus sundaica* and one *Ficus glaberrima*—established epiphytically. Ground‐rooted individuals developed spreading soil root systems rather than aerial strangling roots (Figure 1). In Makmur, seedlings were often found in moist litter and recently flooded soils. In Sangar, figs occurred farther from river margins. Ground‐established figs ranged from recent seedlings to larger individuals, with the numerous small seedlings clearly representing recent natural regeneration.

## 4 Discussion

Our observations show that at least in some instances, and when other tree species are few, strangler figs establish frequently on the ground in burned and open tropical peatlands. Most individuals (97%) germinated without hosts. Strangler figs typically establish as epiphytes on other woody plants (Athreya 1999; Laman 1995) and occasionally colonise rocks, walls, and other structures (Gates 1982; Jim 2014). Past observations indicated that they also establish occasionally on the ground (Zotz et al. 2021; Mo et al. 2022; Berg and Corner 2005; Li et al. 2024).

What enables this terrestrial establishment? Site histories, including the fires in 2013–2014, eliminated many potential host trees, reducing opportunities for epiphytic establishment. The litter and soil conditions appear suitable to provide germination substrates. The presence of recently established seedlings, alongside large individuals, suggests ongoing recruitment.

None of the fig species encountered are narrow endemics; all occur widely across the Sunda region or more broadly across Malesia (Corner 1960; Berg and Corner 2005; GBIF 2024). The capacity for terrestrial establishment under suitable conditions is thus likely to be geographically widespread.

These strangler figs were among the most abundant trees in areas where regeneration of other native species was sparse and planted trees showed poor survival. This abundance likely reflects not only their flexible establishment requirements but also effective animal dispersal, alongside traits including high fecundity and persistence in open conditions. Figs may be especially valuable in restoration and recovery as their fruit attracts seed‐dispersing animals able to bring other tree species into the landscape (Shanahan et al. 2001; Harrison 2005).

The spontaneous regeneration by strangler figs in areas that have proved challenging to restore may accelerate ecological recovery at no additional cost. Aside from the abundant small seeds and broad dispersal, strangler fig species have other characteristics of pioneers, including tolerance of hot, relatively open environments. Densities of these species tend to be lower in old‐growth forests than in disturbed habitats (Harrison et al. 2003; Male and Roberts 2005). They are often abundant in secondary regrowth forests, forest edge environments, and clearings (Patel 1996; Berg and Corner 2005; Mo et al. 2022). They thus appear well able to establish, persist, and grow in many open habitats.

Fig species have considerable potential in forest recovery. The ability of many fig species to establish, grow, and thrive in open environments, their prevalence, and their role in attracting and sustaining seed‐dispersing frugivores (birds and bats) has made them a common choice in various forms of nature‐focused plantings, forest restoration, and other related management choices (Guevara et al. 2004; Cottee‐Jones et al. 2016; Guzmán‐Luna and Martínez‐Garza 2016; Zahawi and Leighton 2018; Elliott et al. 2003).

Restoration strategies for degraded peat swamp forests should consider harnessing the natural regeneration capacity of strangler figs. This would involve protecting naturally established figs and potentially creating and managing conditions to favour their establishment. Due to their resilience and wider ecological relationships, strangler figs are good candidates for use in restoration, and their presence can enhance vegetation density and diversity by introducing seeds of other tree species via dispersal agents.

## Author Contributions

**Sidiq Purwanto:** data curation (lead), formal analysis (lead), investigation (lead), methodology (equal), project administration (equal), writing – original draft (equal). **Muhammad Iqbal:** conceptualization (supporting), investigation (supporting), project administration (supporting), supervision (supporting), writing – review and editing (supporting). **Douglas Sheil:** conceptualization (lead), methodology (equal), supervision (lead), writing – review and editing (lead).

## Conflicts of Interest

The authors declare no conflicts of interest.

## Data Availability

The transect dataset supporting the findings of this study is publicly available in Zenodo at: https://urldefense.com/v3/__https://zenodo.org/records/18663085__;!!N11eV2iwtfs!oRnTSy_r4ysO5hjXRl2D0zEPbnq2c6HZj5O6fGwezU5zirgDAmhCbLtJlQmJFYGpvDxGpTn‐Yz65eOscOnZqhA. The dataset includes species identity, site, measurements, and establishment classification for all recorded tree individuals ≥ 50 cm tall within the study transects.
